# Analytical study of integrating downhole thermoelectric power generation with a coaxial borehole heat exchanger in geothermal wells

**DOI:** 10.1038/s41598-024-51226-0

**Published:** 2024-01-04

**Authors:** Yong Qiao, Kaiyuan Shi, Junrong Liu

**Affiliations:** 1 School of Geosciences, Yangtze University, University Road 111, Caidian Street, Caidian District, Wuhan, 430100 Hubei People’s Republic of China; 2 China Renewable Energy Engineering Institute, Liupukang North Street 2, Xicheng District, Beijing, 100120 People’s Republic of China; 3 Shanghai Hilong Drill Pipe Co.,Ltd, Luodong Road 1825-1, Baoshan District, Shanghai, 200949 People’s Republic of China; 4 School of Petroleum Engineering, China University of Petroleum (East China), Changjiang West Road 66, Huangdao District, Qingdao, 266580 People’s Republic of China

**Keywords:** Energy harvesting, Renewable energy

## Abstract

Geothermal power generation employing Organic Rankine Cycle (ORC) technology is a widely acknowledged and conventional approach for harnessing geothermal energy. In an innovative advancement, we propose a novel design integrating downhole thermoelectric power generation with a coaxial borehole heat exchanger. This design aims to enhance the efficiency and sustainability of geothermal energy utilization. In this innovative design, the geothermal well is divided into two distinct sections: a power generation section and a heat exchanging section, achieved through the implementation of a packer positioned from the uppermost part of the targeted zone. The process involves the injection of cold fluid downhole via an insulated pipe. Subsequently, a portion of the injected fluid is directed to flow in reverse within the casing-tubing annulus above the packer, while another portion circulates into the casing-tubing annulus below the packer before ascending through the tubing. This dual flow mechanism establishes distinct cold and hot sources for the thermoelectric generator, a key feature facilitated by this innovative design. Analytical models detailing of downhole temperature distribution for thermoelectric power have been meticulously developed. A comprehensive case study, focusing on a geothermal well with 3000 m length of power generation section and 500 m heat exchanging section, has been conducted. The results indicate that a significant generating capacity could be achieved with a higher wellhead temperature, and the payback period under different carbon tax scenarios is about 6–8 year. Furthermore, the effects of injection rate, fluid diversion ratio, and casing-tubing configuration on power performance and thermal-electricity efficiency are also discussed. This method not only enables the concurrent harvesting of geothermal energy and power generation but also operates consistently throughout the year. The results thus emphasize the viability and economic feasibility of the proposed approach.

## Introduction

Recently, environmental pollution and extreme climate events have happened frequently. Developing and utilizing geothermal energy is put forward as urgent work and becomes one of the most important measures to solve livelihood issues. Geothermal energy is a clean, renewable, and sustainable energy source. It is estimated that the Earth’s total heat flux is about 44.2 ± 1.0 TW^1^. Geothermal energy will undoubtedly become an important part of new energy in the future.

Recently, geothermal power generation has developed rapidly. According to the statistics, the total installed geothermal power generation capacity in 2015 was 12,647 MWe. The annual electrical generation in 2019 was 92 TWh, approximately 1.0% of global electricity generation^2^. Nevertheless, the world's target by 2050 is to reach 1180 TWh of annual electricity generation from geothermal sources, which is about 2.5–3.1% of global electric demand^3^.

The power generation technology currently available in the geothermal industry depends on the reservoir properties (e.g., geological, geophysical, geochemical, physicochemical, thermodynamic, and others). Dry steam, flash (single, double, and triple), and binary cycle power plants are three types of mature geothermal power generation technologies^4^. Dry steam technology uses the vapor extracted from high- temperature reservoirs (> 240 °C) to produce electricity with a steam turbine and a generator. It is the cheapest geothermal generation process. Flash technology is usually used for liquid–vapor geothermal fluids, where a separation process is needed to generate power. The liquid–vapor separation process can include one, two, or three stages, namely single-, double-, and triple-flash systems, respectively. The single-flash technology is generally used for mixtures with temperature over 210 °C. The double-flash technology can increase both the efficiency of the process and the power generation in relation to the single-flash technology. The triple-flash technology is designed to utilize the energy available in the brine coming from the double flash cycle. The binary cycle system is usually used for the geofluid produced from liquid-dominated reservoirs with temperatures lower than 200 °C. Due to the low temperature of the geofluid, a working fluid (which has a lower boiling point, e.g., n-isobutane, n-isopentane, and pentane) is used to vaporize the thermal energy in the geofluid to produce electricity with Organic Rankine Cycle (ORC) or Kalina Cycle^5,6^.

Many studies have focused on use of the borehole exchangers to extract geothermal heat^7^. It only extracts heat without producing mass (fluid) from the reservoir, so there are many advantages, such as reducing construction costs by eliminating surface facilities and dedicated injectors for brine disposal, avoiding surface subsidence, corrosion, and scaling problems^8^. Different designs of downhole heat exchangers were developed to harvest heat by different heat-transfer mechanisms, such as conduction^9^, natural convection^7^, and forced convection^10^. Morita et al.^11^ proposed a Downhole Coaxial Heat Exchanger (DCHE) system combined with binary or Kalina cycles for the exploitation of geothermal energy. Their results showed that 70 kW of power generation might be possible with a DCHE 2,000 m deep. Feng et al.^10^ used a DCHE to enhance forced convention driven by a downhole pump. Their case study showed that utilizing such a configuration in a horizontal well could produce about 350 kW power even after 30 years of operation. Alimonti and Soldo^12^ simulated the heat extracted by optimizing the geometry of the wellbore heat exchanger. For a well between 5800 and 6100 m deep with a temperature of about 160–170 °C, the net electric power of 134 kW could be produced with the ORC technology. Noorollahi et al.^13^ studied the power generation from an abandoned geothermal well with a depth of 3176 m by circulating fluid in a coaxial double-pipe heat exchanger and using ammonia and isobutene as working fluids in a binary cycle. The maximum net powers from the well are 270 and 181 kW for isobutane and ammonia with a mass flow rate of 12 kg/s respectively. Alimonti et al.^14^ compared the thermal-to-electric conversion efficiency between the Organic Ranking Cycle (ORC) plant and the Stirling motor with a wellbore heat exchanger. With the same working parameters, the net electric powers are 121 kW for ORC and 152 kW for Stirling motor. Akhmadullin and Tyagi^15^ proposed to harvest geothermal energy in a horizontal well with coupled production and injection sections utilizing a downhole heat exchanger. A pump was used to circulate hot brine through the downhole heat exchanger between the production and injection sections and produce no fluid on the surface. In one single lateral well, the net electric power of 160 kW could be produced with CO_2_ working fluid at 7000 m well depth.

A lot of geothermal resources found in the world belong to the low-temperature category, which are usually used in combined heat and power plants. For water with a temperature below 100 °C, binary (Organic rankine cycle) power plants are usually used to generate electricity. Besides this technology, the thermoelectric generator (TEG) technology is considered a new way to produce power for low-temperature geothermal resources^16^. Comparing with the conventional methods of generating electricity from geothermal energy using ORC technology, the thermoelectric method can directly convert heat into electricity by the Seebeck effect and has numerous advantages, including direct energy conversion, no moving parts and no working fluids inside the thermoelectric generator, no maintenance and no extra costs, no land needed, superior scalability, a long lifespan, independent of the hour of the day and season, noiseless operations, high reliability, and environmental friendliness. In addition, it has a wider choice of thermal sources. It can utilize both high- and low-quality heat to generate power, while the ORC technology works ineffectively for low-quality heat^16–21^.

Li et al.^16^ built a power generator with 96 TEG modules in the laboratory to validate power generation with geothermal fluid. An output power of approximately160W has been generated with a temperature difference of 80 °C. The instantaneous thermal-electricity conversion efficiency of the TEG system reached 4.5% at an inlet temperature of about 95 °C on the hot side and a temperature of 30 °C on the cold side. Chet et al.^19^ proposed a thermosiphon heat exchanger with a number of thermoelectric cells (TECs) for power generation from geothermal energy. The thermosiphon heat exchanger was inclined on the ground and vertically extended into the downhole to extract geothermal energy. The liquid under vacuum boils and becomes vapor with phase change. The heat carried by vapor ascends to the heat exchanger and transfers heat to the thermoelectric cells on the ground. Then it realizes power generation under a certain temperature difference. The proposed system is able to produce 1800 kW of electric power with a thermal-electricity efficiency of 8–﻿9%. The cost per unit output power for the system proposed is around $1/watt to $1.5/watt. Depending on the cooling water outlet temperature ranging from 15 to 100 °C, the number of TECs needed ranges from 350,000 to 520,000, or equivalent to a total heat exchanger length of 350–520 m. Wang et al.^22^ studied downhole power generation in oil wells with a 20 m long of thermoelectric generator. 9,848W of electric power and 4.7% of thermal-electricity conversion efficiency were obtained. Bitschi^23^ studied the feasibility of using TEG to exploit geothermal energy with analytical model and a numerical approach. In his work, a one-dimensional, steady- state, numerical model for a counterflow type TEG was built, and the effects of the temperature changes at heat exchanger and the temperature difference through thermoelectric elements in the thermoelectric generator on power performance were analyzed.

Date et al.^18^ gave a detailed review of thermoelectric power generation systems for small-scale to medium-scale power generation. Liu^24^ presented a new design for an electricity generator based on thermoelectric effects, where the thermoelectric tubes with a one-meter length and different thicknesses were arranged in parallel. It could generate a megawatt of power with several hundred to several thousand thermoelectric tubes using heat resources with10K temperature differences. Liu et al.^25^ developed the segmented thermoelectric leg for the fabrication of multistage thermoelectric generators. Compared with cascaded TEGs, segmented TEGs are compact and high in power density, which is preferable for the application of medium- or high-temperature power generation. Kim et al.^26^ investigated the waste heat recovery performance of a direct contact thermoelectric generator on a diesel engine. The results showed that a 10 K decrease in the coolant temperature yields a smaller increase (0.25%) in the conversion efficiency for the engine, while a 20 K decrease causes a larger increase (15%) in the conversion efficiency. However, these attempts are all harvesting heat to generate electricity on a smaller scale, and very limited attention has been paid to generating electricity in downhole with more than thousands of feet length.

The aim of this work is to find an alternative method in which electric power may be generated in the downhole with thermoelectric technology and coaxial borehole heat exchanger technology in geothermal wells, which is different from traditional ORC or Kalina technology. The coaxial borehole heat exchanger recovers heat from the surrounding formation, and transfers the heat to thermoelectric modules to generate power in the downhole. Mathematical models are developed to study the downhole temperature distributions related to power generation. Then, a case study with cost–benefit analysis will be conducted to validate the potential of downhole thermoelectric power generation in geothermal wells under different carbon taxes. Finally, the factors that affect the performance of downhole thermoelectric power generation are investigated, and the optimum operational parameters are determined.

## Thermoelectric generator

**Figure 1:**
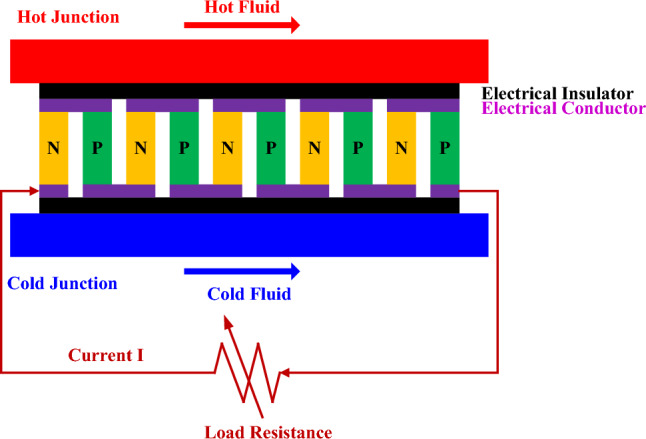
Schematic of a thermoelectric generator.

A thermoelectric generator (TEG) is a device that directly converts a heat gradient into electricity through the Seebeck effect, which is the phenomenon that electric current is induced by a temperature difference in an electrical conductor. A TEG is usually made up of ceramic substrates, electrical insulators, electrical conductors, and many N-type and P-type semiconductors with high Seebeck cofficients that are alternately connected in series electrically and in parallel thermally, as shown in Fig. 1. The Seebeck coefficient is the generated voltage difference per degree of temperature difference of a material. It is material- and temperature-dependent, with negative values for the N-type and positive values for the P-type semiconductors.

Electrical resistivity and thermal conductivity are two other important parameters when selecting materials for thermoelectric power generation and maintaining a high heat gradient. Seebeck coefficient, electrical resistivity, and thermal conductivity are combined into the figure of merit, ${\text{Z}}$, which is a measure of the heat-to-electricity conversion efficiency. Usually, the dimensionless figure of merit,${\text{ZT}}$, which is the product of the figure of merit by the average temperature, is used to compare the properties of materials. The higher the ${\text{ZT}}$ is, the higher the heat-to-electricity conversion efficiency will be^27–29^.

For a thermoelectric pair with cross-areas of ${{\text{A}}}_{{\text{N}}}$ and ${{\text{A}}}_{{\text{P}}}$ for N-type and P-type semiconductors, with the same length of ${\text{L}}$, the thermal conductance ${\text{K}}$, and electrical resistance ${\text{R}}$, could be expressed as^27,30^,

1 $$K={K}_{n}+{K}_{p}={k}_{p}\frac{{A}_{p}}{{L}_{np}}+{k}_{n}\frac{{A}_{n}}{{L}_{np}}$$

2 $$R={R}_{n}+{R}_{p}={\sigma }_{p}\frac{{L}_{np}}{{A}_{p}}+{\sigma }_{n}\frac{{L}_{np}}{{A}_{n}}$$

where, ${K}_{n}$ and ${K}_{p}$ are the thermal conductance of N-type and P-type semiconductors, respectively, W/K; ${k}_{n}$ and ${k}_{p}$ are the thermal conductivity of N-type and P-type semiconductors, respectively, W/(m K); ${R}_{n}$ and ${R}_{p}$ are the electric resistance of N-type and P-type elements in a TEG, respectively, Ω; ${\sigma }_{n}$ and ${\sigma }_{p}$ are the electrical resistivity of N-type and P-type semiconductors, respectively, Ω m; ${A}_{n}$ and ${A}_{p}$ are the cross-section areas for N-type and P-type semiconductors, m^2^; ${L}_{np}$ is the length of semiconductors, m.

The figure of merit (Z) is defined as,

3 $$Z=\frac{{\alpha }^{2}}{k\sigma }$$

where, $k$ is the thermal conductivity of a pair of N-type and P-type semiconductors, W/(m K); $\sigma$ is the electrical resistivity of a pair of N-type and P-type semiconductors, Ω m; and $\alpha$ is the overall Seebeck coefficients of a pair of N-type and P-type semiconductors, V/K.

## System design of downhole thermoelectric power generation system with coaxial borehole heat exchanger

**Figure 2:**
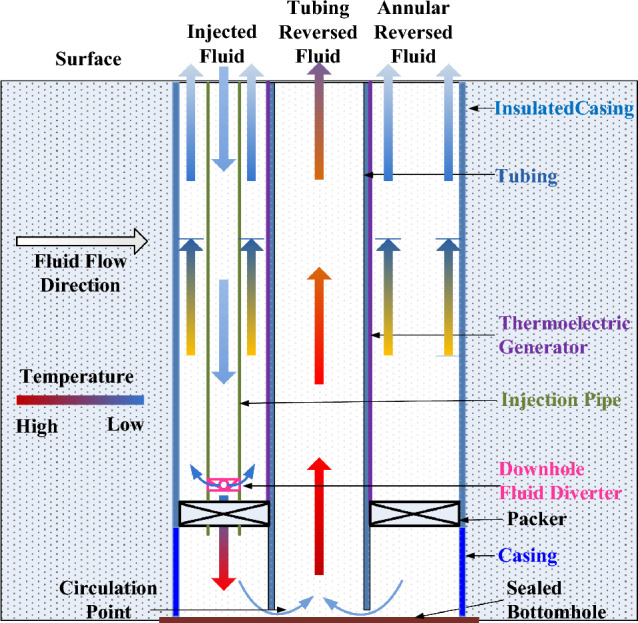
Schematic of downhole thermoelectric power generation design with a coaxial borehole heat exchanger in a geothermal well.

A Downhole coaxial heat exchanger is a way to extract geothermal energy without producing geothermal fluid^11,13,31,32^. The extracted heat from the working fluid is usually used for direct utilization or ORC power generation. Recently, power generation with thermoelectric technology has been proposed to harvest and utilize geothermal energy in downholes^22,33–36^. Combining with a coaxial borehole heat exchanger, a new design of downhole thermoelectric power generation in geothermal wells is presented (Fig. 2).

In the proposed design, the well bottom is sealed completely, and the casing is lowered to the well bottom to prevent leakage between the formation and borehole. Tubing is run into casing and downed to right above the sealed bottom. A packer will be set at the top of production zone to isolate the casing-tubing annulus. An injection pipe (vaccum-insulated steel pipe^37^) assembled with a downhole fluid diverter will be run into the casing-tubing annulus and just pass through the packer. The downhole fluid diverter is located just above the packer and is used to adjust fluid flowing into the casing-tubing annulus above and below the packer. The outer surfaces of the tubing above the packer are fully covered with thermoelectric modules. Cold fluid will be continuously injected downward in the injection pipe from the surface. When the cold fluid flows pass the downhole fluid diverter, part of the cold fluid will be diverted and enters into the casing-tubing annulus above the packer; the other part will continuously flow downwards through the packer and enters the casing-tubing annulus below the packer, which will reverse back at the circulation point due to the sealed bottomhole. The diverted cold fluid at the downhole fluid diverter flows upwards along the casing-tubing annulus above the packer and provides cold sources for TEGs. While the other part of the cold fluid continuously flows downwards along the casing-tubing annulus below the packer, it adsorbs heat from the surrounding formation gradually and becomes high-temperature fluid at the bottom of the well. At the circulation point, the heated fluid flows upwards in tubing to the surface and provides heat sources for TEGs. At the same well depth, the temperature of upward flowing fluid in tubing is higher than that of upward flowing fluid in casing-tubing annulus. Once the system achieves a stable temperature difference between the tubing and casing-tubing annulus by continuously injecting and circulating the cold fluid, electricity will be generated as a response to the applied temperature gradient, and the produced electricity could be transmitted to the surface and input to the local grid. In general, the proposed design includes two sections: one is the heat harvesting section located below the packer, which harvests heat from the surrounding hot formation with a coaxial borehole heat exchanger; and the other is the thermoelectric power generation section located above the packer, which produces power by measuring the temperature difference across both sides of TEGs.

In order to create a temperature difference as large as possible across both sides of TEGs, the upper casing above the packer is coated with insulation materials (such as nano-SiO_2_ aerogel^37^) to reduce heat—transfer from the surrounding formation to the fluid flowing in casing-tubing annulus, while the lower casing below the packer has a good heat conductivity to harvest enough geothermal energy from the deeper formation to heat the injected cold fluid in it. The tubing and injection pipe surfaces are also coated with insulation materials.

Compared to TEG applications in other industries, downhole power generation represents a large-scale application, especially given the depth of geothermal wells. Suzuki and Tanaka^38^ pointed out that the output power has a decreasing variation after first increasing with the increased length of the TEGs. Montecucco et al.^39^ disclosed that connecting thermoelectric generators in series is better for electrical system efficiency than in parallel when the temperature differences remain constant. Therefore, segmented TEGs are used and connected in series when considering the longer depth of the well.

## Mathematical model for downhole temperature and power generation

### Downhole temperature model

To assess the performance of the proposed downhole power generation design, it is necessary to know the temperature distributions along the tubing, the casing-tubing annulus, and both sides of TEGs. The assumptions are made: (1) Injection pipe is perfectly insulated; the packer length and the injection pipe length extending out of the packer are ignored that is, the inlet temperatures of the injected cold fluid at the casing-tubing annulus above the packer and below the packer are the same as the inlet temperature at surface; (2) The heat transfer between formation and wellbore is in a steady state; (3) Temperature drops across both tubing and casing walls are neglected due to the high thermal conductivity of metals as well as the small thickness of the walls; (4) Thermoelectric elements in TEG are identical, and their geometric configurations are in the optimum form; (5) External heat- transfer irreversibility between the thermoelectric devices and the heat reservoirs are neglected; (6) Seebeck coefficient, thermal conductance, electrical resistance, and figure of merit of the thermoelectric devices are independent of temperature in the range of studied temperatures; (7) Fluid in tubular flows in one-dimension axial direction and heat conducts in one-dimension radical direction.

**Figure 3:**
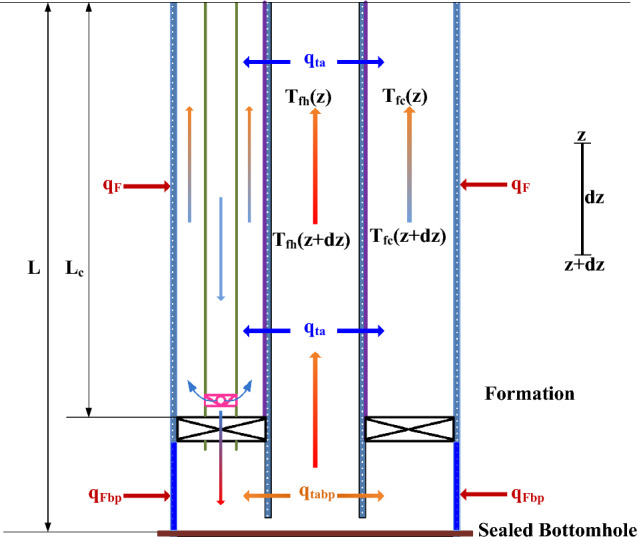
Schematic of heat balance for tubular and formation.

Heat exchanges among tubing fluid, casing-tubing annular fluid, coaxial borehole heat exchanger, and surrounding formation result in temperature differences on both sides of TEGs. To obtain temperature distributions in the casing-tubing annulus, tubing, and coaxial borehole heat exchanger, a heat balance over an element of length, ${\text{dz}}$, which is treated as a control volume at a distance of ${\text{dz}}$ from the surface where $z$ equals to zero, was built. Here ${\text{z}}$ is positive in the downward direction. The schematic heat balance for the tubular and formation is depicted in Fig. 3.

#### Below packer (heat harvesting section)

The flow conduits below the packer work as a coaxial borehole heat exchanger. In the casing-tubing annulus below the packer, the injected fluid is in direct contact with the casing inner wall. Assuming the casing is cemented with the rock in good condition, the heat transfer between the rock and the casing happens by conduction, and between the casing wall and the reversed fluid in casing-tubing annulus by convection. The convection into the formation is ignored. In the tubing, the reversed fluid from the circulation point flows up and enters the thermoelectric power generation section; the heat transfer occurs only through the tubing wall^12,40,41^. In this study, water was selected as the working fluid. For the fluid flows from depth of ${\text{z}}$ to $({\text{z}}+{\text{dz}})$ in casing-tubing annulus and from depth of $({\text{z}}+{\text{dz}})$ to ${\text{z}}$ in tubing below the packer, energy balance equations could be established accordingly.

4 $${q}_{fhbp}\left(z+dz\right)={q}_{fhbp}\left(z\right)+{q}_{tabp}$$

5 $${q}_{fcbp}\left(z+dz\right)={q}_{fcbp}\left(z\right)+{q}_{tabp}+{q}_{Fbp}$$

where ${q}_{fhbp}$ is the heat of the reversed fluid in tubing below the packer, J; ${q}_{fcbp}$ the heat of the injected fluid in casing-tubing annulus below the packer, J; ${q}_{tabp}$ is the heat flow from the tubing to the casing-tubing annulus, J; ${q}_{Fbp}$ is the heat flow from the surrounding formation to the casing-tubing annulus, J.

Based on the heat-transfer theory, the heat balance for fluid flowing in the tubing and in the casing-tubing annulus below the packer is respectively given by:

6 $${q}_{fhbp}\left(z+dz\right)-{q}_{fhbp}\left(z\right)={c}_{w}{w}_{injbp}\left[{T}_{fhbp}\left(z+dz\right)-{T}_{fhbp}\left(z\right)\right]$$

7 $${q}_{fcbp}\left(z+dz\right)-{q}_{fcbp}\left(z\right)={c}_{w}{w}_{injbp}\left[{T}_{fcbp}\left(z+dz\right)-{T}_{fcbp}\left(z\right)\right]$$

where ${c}_{w}$ is the water specific heat capacity, J/(kg.K); ${w}_{injbp}$ is the mass of the injected fluid in the casing-tubing annulus below the packer, kg; ${T}_{fhbp}$ is the fluid temperature in the tubing below the packer, K; ${T}_{fcbp}$ is the fluid temperature in the casing-tubing annulus below the packer, K.

Heat flow, ${q}_{Fbp}$, from surrounding formation to the casing-tubing annulus below the packer is given below,

8 $${q}_{Fbp}=2\pi {r}_{c}{U}_{abp}\left({T}_{wb}\left(z\right)-{T}_{fcbp}\left(z\right)\right)dz$$

Heat flow, ${q}_{ta}$, from tubing to casing-tubing annulus below the packer is given by:

9 $${q}_{tabp}= 2\pi {r}_{t}{U}_{tbp}\left({T}_{fhbp}(z)-{T}_{fcbp}(z)\right)dz$$

where ${r}_{c}$ is the casing radius, m; ${r}_{t}$ is the tubing radius, m; ${T}_{wb}$ is the temperature at wellbore/formation interface, K; ${U}_{abp}$ is the overall heat-transfer coefficient of casing-tubing annulus below the packer, which depends on the resistances to heat flow through casing-tubing annular fluid, casing metal, and cement, W/(m K); ${U}_{tbp}$ is the overall heat-transfer coefficient of tubing the packer, which depends on the resistances to heat flow through the tubing fluid and tubing metal, W/(m K). ${U}_{abp}$ and ${U}_{tbp}$ can be calculated by many methods^42,43^.

Assuming that the temperature at the wellbore/formation interface along the vertical direction changes linearly, that is:

10 $${T}_{wb}={T}_{surface}+{g}_{G}z$$

where, ${T}_{surface}$ is the surface temperature of the wellbore/formation interface, K; ${g}_{G}$ is the geothermal gradient, K/m; $z$ is the well depth from surface, m.

Letting,

$$A = \frac{{c_{w} w_{injbp} }}{{2\pi r_{c} U_{a} }}\quad B = \frac{{c_{w} w_{injbp} }}{{2\pi r_{t} U_{t} }}$$

Simplifying these equations based on the assumptions of incompressible, single-phase fluid, and the following equations can be obtained,

11 $$A\frac{d{T}_{fcbp}}{dz}=\left({T}_{wb}-{T}_{fcbp}\right)-\frac{A}{B}({T}_{fcbp}-{T}_{fhbp})$$

12 $$\frac{d{T}_{fhbp}}{dz}=\frac{{T}_{fhbp}-{T}_{fcbp}}{B}$$

The boundary conditions could be found to be that the temperature of the reversed fluid at the tubing inlet is equal to the temperature of the injected fluid in the casing-tubing annulus at the circulation point, and the temperature of the injected fluid at the outlet of the injection pipe is known. Here, we assume that the depth of the tubing inlet (circulation point) is the same as the well bottom. Circulation point at the tubing inlet and injection pipe outlet is expressed as:

13 $$z = {\text{L}}\quad T_{fhbp} = T_{fcbp}$$

14 $$z = L_{c} \quad T_{fcbp} = T_{inj}$$

where ${\text{L}}$ is the depth of the tubing inlet or the circulation point or the well bottom, m; ${L}_{c}$ is the depth of cold fluid entering into the casing-tubing annulus below the packer or the depth of the downhole fluid diverter or the depth of injection pipe outlet, m; ${T}_{inj}$ is the temperature of injected liquid at the surface, K.

Applying boundary conditions, the temperature distribution along tubing and casing-tubing annulus can be solved and expressed as,

15 $${T}_{fhbp}\left(z\right)=m{e}^{{\lambda }_{1}z}+n{e}^{{\lambda }_{2}z}+{T}_{surface}+{g}_{G}z+B{g}_{G}+{T}_{wb}\left({L}_{c}\right)$$

16 $${T}_{fcbp}\left(z\right)=\left(1-{\lambda }_{1}B\right)m{e}^{{\lambda }_{1}z}+\left(1-{\lambda }_{2}B\right)n{e}^{{\lambda }_{2}z}+{T}_{wb}\left({L}_{c}\right)+{T}_{surface}+{g}_{G}z$$

where, ${T}_{wb}\left({L}_{c}\right)$ is the formation temperature at the packer, K.

In Eqs. (15) and (16), ${\lambda }_{1}$, ${\lambda }_{2}$, *m* and *n* are all constants, given as follows.

$${\lambda }_{1}=-\frac{1}{2A}+\frac{1}{2A}\sqrt{1+\frac{4A}{B}}$$

$${\lambda }_{2}=-\frac{1}{2A}-\frac{1}{2A}\sqrt{1+\frac{4A}{B}}$$

$$m=-\frac{\left({T}_{inj}-{T}_{wb}\left({L}_{c}\right)\right){\lambda }_{2}{e}^{{\lambda }_{2}\left(L-{L}_{c}\right)}+{g}_{G}(1-{\lambda }_{2}B)}{{\lambda }_{1}{e}^{{\lambda }_{1}\left(L-{L}_{c}\right)}\left(1-{\lambda }_{2}B\right)-{\lambda }_{2}{e}^{{\lambda }_{2}\left(L-{L}_{c}\right)}\left(1-{\lambda }_{1}B\right)}$$

$$n=\frac{\left({T}_{inj}-{T}_{wb}\left({L}_{c}\right)\right){\lambda }_{1}{e}^{{\lambda }_{1}\left(L-{L}_{c}\right)}+{g}_{G}(1-{\lambda }_{1}B)}{{\lambda }_{1}{e}^{{\lambda }_{1}\left(L-{L}_{c}\right)}\left(1-{\lambda }_{2}B\right)-{\lambda }_{2}{e}^{{\lambda }_{2}\left(L-{L}_{c}\right)}\left(1-{\lambda }_{1}B\right)}$$

#### Above packer (power generation section)

For the fluid heated in the casing-tubing annulus below the packer and flowing upward in the tubing, it enters at the depth of $({\text{z}}+{\text{dz}})$ and leaves at ${\text{z}}$ with heat convection towards the casing-tubing annulus above the packer; and for the fluid flowing upward in the casing-tubing annulus above the packer, the energy balance involves heat transfer from the tubing to the casing-tubing annulus above the packer and heat-transfer from surrounding formation^40,41^. Therefore, energy balance equations could be established in tubing and casing-tubing annulus, accordingly.

17 $${q}_{fh}\left(z\right)={q}_{fh}\left(z+dz\right)-{q}_{ta}$$

18 $${q}_{fc}\left(z\right)={q}_{fc}\left(z+dz\right)+{q}_{ta}+{q}_{F}$$

where ${q}_{fh}$ is the heat of the reversed fluid in tubing above the packer, J; ${q}_{fc}$ the heat of the reversed fluid in casing-tubing annulus above the packer, J; ${q}_{ta}$ is the heat flow from tubing to casing-tubing annulus above the packer, J; ${q}_{F}$ is the heat flow from surrounding formation to casing-tubing annulus above the packer, J.

Based on the heat transfer theory, the heat balance for fluid flowing in tubing and in the casing-tubing annulus above the packer is respectively given by:

19 $${q}_{fh}\left(z+dz\right)-{q}_{fh}\left(z\right)={c}_{w}{w}_{injbp}\left[{T}_{fh}\left(z+dz\right)-{T}_{fh}\left(z\right)\right]$$

20 $${q}_{fc}\left(z+dz\right)-{q}_{fc}\left(z\right)={c}_{w}{w}_{inj}\left[{T}_{fc}\left(z+dz\right)-{T}_{fc}\left(z\right)\right]$$

where ${c}_{w}$ is the water specific heat capacity, J/(kg.K); ${w}_{inj}$ is the mass of the reversed fluid in the casing-tubing annulus above the packer, kg; ${T}_{fh}$ is the fluid temperature in the tubing above the packer, K; ${T}_{fc}$ is the fluid temperature in the casing-tubing annulus above the packer, K.

Heat flow, ${q}_{F}$, from the surrounding formation to the casing-tubing annulus above the packer is given by:

21 $${q}_{F}=2\pi {r}_{c}{U}_{a}\left({T}_{wb}\left(z\right)-{T}_{fc}\left(z\right)\right)dz$$

Heat flow, ${q}_{ta}$, from the tubing to the casing-tubing annulus above the packer is given by:

22 $${q}_{ta}= 2\pi {r}_{t}{U}_{t}\left({T}_{fh}(z)-{T}_{fc}(z)\right)dz$$

where ${r}_{c}$ is the casing radius, m; ${r}_{t}$ is the tubing radius, m; ${T}_{wb}$ is the temperature at wellbore/formation interface, K; ${U}_{a}$ is the overall heat-transfer coefficient of casing-tubing annulus above the packer, which depends on the resistances to heat flow through annular fluid, insulation material on the casing surface, casing metal, and cement, W/(m K); ${U}_{t}$ is the overall heat-transfer coefficient of tubing above the packer, which depends on the resistances to heat flow through tubing fluid, tubing metal, and insulation material on the tubing surface, W/(m K).

Simplifying these equations based on the assumptions of incompressible and single-phase fluids, the following equations can be obtained:

23 $$\frac{d{T}_{fh}}{dz}=\frac{2\pi {r}_{t}{U}_{t}}{{c}_{w}{w}_{injbp}}({T}_{fh}-{T}_{fc})$$

24 $$\frac{d{T}_{fc}}{dz}=-\frac{1}{{c}_{w}{w}_{inj}}[2\pi {r}_{t}{U}_{t}\left({T}_{fh}-{T}_{fc}\right)+2\pi {r}_{c}{U}_{a}\left({T}_{surface}+{g}_{G}z-{T}_{fc}\right)]$$

The boundary conditions could be found that the outlet temperature of the coaxial borehole heat exchanger section is equal to the inlet temperature of the thermoelectric power generation section, and the inlet temperature of the casing-tubing annulus above the packer is equal to the temperature of the injected fluid. Then,

25 $$z = L_{c} \quad T_{fh} = T_{fhbp} \left( {L_{c} } \right)$$

26 $$z = L_{c} \quad T_{fc} = T_{inj}$$

where ${T}_{fhbp}({L}_{c})$ is the outlet temperature of the coaxial borehole heat exchanger section, K; ${T}_{inj}$ is the temperature of the injected liquid, K.

By applying boundary conditions, the temperature distributions along tubing and casing-tubing annulus can be solved and expressed as,

27 $${T}_{fh}\left(z\right)=m{e}^{{\lambda }_{1}z}+n{e}^{{\lambda }_{2}z}+{T}_{surface}+{g}_{G}(z+\xi )$$

28 $${T}_{fc}\left(z\right)=\left(1-{\lambda }_{1}B\right)m{e}^{{\lambda }_{1}z}+\left(1-{\lambda }_{2}B\right)n{e}^{{\lambda }_{2}z}+{T}_{surface}+{g}_{G}(z+\xi -B)$$

In Eqs. (27) and (28), *C, D, E*,$\xi$, ${\lambda }_{1}$, ${\lambda }_{2}$, *m* and *n* are all constants, given as follows.

$$C = \frac{{c_{w} w_{inj} }}{{2\pi r_{c} U_{a} }}\quad D = \frac{{c_{w} w_{t} }}{{2\pi r_{t} U_{t} }}\quad E = \frac{{c_{w} w_{inj} }}{{2\pi r_{t} U_{t} }}$$

$$\upxi =\frac{CD+DE+CE}{E}$$

$${\lambda }_{1}=\frac{CD+DE+CE+\sqrt{{(CD+DE+CE)}^{2}-4CD{E}^{2}}}{2CDE}$$

$${\lambda }_{2}=\frac{CD+DE+CE-\sqrt{{(CD+DE+CE)}^{2}-4CD{E}^{2}}}{2CDE}$$

$$m=\frac{\left(1-{\lambda }_{2}D\right)\times \left({T}_{fhbp}({L}_{c})-{T}_{surface}-{g}_{G}\left({L}_{C}+\xi \right)\right)-({T}_{inj}-{T}_{surface}-{g}_{G}\left({L}_{C}+\xi -D\right))}{D\times ({\lambda }_{1}-{\lambda }_{2}){e}^{{\lambda }_{1}{L}_{C}}}$$

$$n=\frac{\left(1-{\lambda }_{1}D\right)\times \left({T}_{fhbp}({L}_{c})-{T}_{surface}-{g}_{G}\left({L}_{C}+\xi \right)\right)-({T}_{inj}-{T}_{surface}-{g}_{G}\left({L}_{C}+\xi -D\right))}{D\times ({\lambda }_{2}-{\lambda }_{1}){e}^{{\lambda }_{2}{L}_{C}}}$$

### Electrical power generation

The thermoelectric modules are attached tightly on the outer surface of the tubing, and their geometric configurations are in their optimum state. When the flowing temperature in the tubing and casing-tubing annulus is known, then the temperature on the hot and cold surfaces of TEGs can be deduced from heat transfer theory^38^ and expressed as follows

29 $${T}_{mh}={T}_{fh}-\frac{2\pi {r}_{pn}{U}_{t}}{{h}_{t}}\left({T}_{fh}-{T}_{fc}\right)$$

30 $${T}_{mc}={T}_{fc}+\frac{2\pi {r}_{pn}{U}_{t}}{{h}_{c}}\left({T}_{fh}-{T}_{fc}\right)$$

where ${T}_{mh}$ is the temperature on the hot surface of TEG, K; ${T}_{mc}$ is the temperature on the cold surface of TEG, K; ${r}_{pn}$ is the radius after the thermoelectric modules are attached on the external surface of the tubing, m; ${h}_{t}$ is the convective heat-transfer coefficient between the produced fluid and the tube, W/(m K); ${h}_{c}$ is the convective heat-transfer coefficient between the injected fluid and the case, W/(m K).

According to the principle of the TEG, the produced voltage depends on the temperature difference and Seebeck coefficient, as given by^44–46^:

31 $$e=\alpha \left({T}_{mh}-{T}_{mc}\right)$$

where $e$ is the voltage of a thermoelectric element, V.

For an element of length (${\text{dz}}$), the temperature along each side of TEG stays constant. Then the total voltage, $E$, can be integrated along the wellbore and given by:

32 $$E={\int }_{0}^{L}edz={n}_{\phi }{n}_{x}\alpha {\int }_{0}^{L}\left({T}_{mh}\left(z\right)-{T}_{mc}\left(z\right)\right)dz$$

where ${n}_{\phi }$ and ${n}_{x}$ are the number of thermoelectric pairs in a circumferential circulation and the number density of thermoelectric pairs in the axial direction, respectively, dimensionless.

When the thermoelectric module is applied to a certain temperature gradient, electric power is produced, which is defined as,

33 $$P={I}^{2}{R}_{L}$$

where ${R}_{L}$ is the external electric resistance, Ω; $I$ is the current flowing through the circuit, A.

The current, $I$, is given as,

34 $$I=\frac{E}{R+{R}_{L}}$$

where $R$ is the internal electric resistance of TEG, Ω.

By Combining Equations. (33) with (34), then,

35 $$P=\frac{{E}^{2}}{2R+{R}_{L}+\frac{{R}^{2}}{{R}_{L}}}$$

where ${\text{P}}$ is the output power, W.

The maximum power output is obtained when the external electric resistance is equal to the internal electric resistance of the TEG^47–50^, this gives,

36 $${P}_{max}=\frac{{E}^{2}}{4R}=\frac{{E}^{2}}{4\left({R}_{n}+{R}_{p}\right)}$$

where ${P}_{max}$ is the maximum output power, W.

The power generated by a downhole thermoelectric power generation system is the sum of the powers generated by all of the segments^51^. The output power of the *ith* segment with a length of ${L}_{i}$ is:

37 $${P}_{i}=\frac{{n}_{\phi }{n}_{x}{L}_{i}[{\alpha \left({T}_{mh(i)}-{T}_{mc(i)}\right)]}^{2}}{4\left({R}_{n}+{R}_{p}\right)}$$

where ${L}_{i}$ is the length of *ith* segment, m.

Then, the total output power in a well is given by:

38 $${P}_{t}=\sum_{i=1}^{{n}_{teg}}{P}_{i}$$

where ${n}_{teg}$ is the number of the thermoelectric generator segments.

By considering the Seebeck effect, the thermal power input to the hot side in the *ith* segment, ${Q}_{Hi}$, and the thermal power output from the cold side in the *ith* segment, ${Q}_{Ci}$, are separately given by^48,52^,

38 $${Q}_{Hi}=K\left({T}_{mh(i)}-{T}_{mc(i)}\right)+\alpha {T}_{mh(i)}I-\frac{1}{2}{I}^{2}R$$

40 $${Q}_{ci}=K\left({T}_{mh(i)}-{T}_{mc(i)}\right)+\alpha {T}_{mc(i)}I+\frac{1}{2}{I}^{2}R$$

where $K$ is the thermal conductance of TEG, W/(m K).

The efficiency of TEG can be defined as,

41 $$\upeta =\frac{{P}_{t}}{\sum {Q}_{Hi}}$$

### Required pumping power

In our proposed design, a surface pump is used to inject and circulate the cold fluid in the injection pipe, casing-tubing annulus, coaxial borehole heat exchanger, and tubing. To attain the required pumping power, the pressure losses in the injection pipe, casing-tubing annulus, coaxial borehole heat exchanger, and tubing should be determined. According to single-phase flow theory, the pressure losses in flow conduits above the packer can be calculated by:

42 $$\Delta {{\text{P}}}_{{\text{ut}}}=\mathrm{\rho g}\Delta {\text{z}}-\Delta {{\text{P}}}_{fut} upward$$

43 $$\Delta {{\text{P}}}_{ua}=-\mathrm{\rho g}\Delta {\text{z}}-\Delta {{\text{P}}}_{fua} upward$$

44 $$\Delta {{\text{P}}}_{dip}=\mathrm{\rho g}\Delta {\text{z}}-\Delta {{\text{P}}}_{fdip} downward$$

where $\Delta {{\text{P}}}_{u{\text{t}}}$ is the pressure loss in tubing above the packer, Pa;$\Delta {{\text{P}}}_{{\text{u}}a}$ is the pressure loss in casing-tubing annulus above the packer, Pa; $\Delta {{\text{P}}}_{dip}$ is the pressure loss in injection pipe, Pa; $\uprho$ is the fluid density, kg/m^3^; ${\text{g}}$ is the gravitational acceleration, m/s^2^; $\Delta {\text{z}}$ is the step in length of pipe, m; $\Delta {{\text{P}}}_{fut}$ is the pressure loss due to friction in tubing, Pa;$\Delta {{\text{P}}}_{fua}$ is the pressure loss due to friction in casing-tubing annulus, Pa; $\Delta {{\text{P}}}_{fdip}$ is the pressure loss due to friction in injection pipe, Pa. Friction losses could be calculated using the universal relation,

45 $$\Delta {{\text{P}}}_{f}=\uplambda \frac{\Delta {\text{Z}}}{D}\rho \frac{{v}^{2}}{2}$$

where $\uplambda$ is the friction factor, dimensionless; $D$ is the diameter of the conduit, m; $v$ is the velocity of the fluid, m/s. The friction factor can be calculated by multiple methods^53,54^.

The pressure losses in coaxial borehole heat exchanger can also be calculated according to Eqs. (42)–(44).

With the known pressure differences, the required pumping power can be obtained.

45 $${P}_{pump}={Q}_{inj}\left[\Delta {{\text{P}}}_{dip}+\omega \Delta {{\text{P}}}_{ua}+\left(1-\omega \right)\left(\Delta {{\text{P}}}_{ut}+\Delta {{\text{P}}}_{dc}+\Delta {{\text{P}}}_{uc}\right)\right]$$

where, ${P}_{pump}$ is the required pumping power, W; ${Q}_{inj}$ is the injection rate at surface, m^3^/s; $\Delta {{\text{P}}}_{dc}$ is the pressure loss in the downward coaxial borehole heat exchanger (casing-tubing annulus below the packer), Pa; $\Delta {{\text{P}}}_{uc}$ is the pressure loss in the upward coaxial borehole heat exchanger (tubing below the packer), Pa; $\omega$ is the ratio of the flow rate entering the casing-tubing annulus above the packer to the total injection rate.

Assuming the pumping power is supplied by the produced power from the same well, then the net power is given by:

46 $${P}_{net}={P}_{t}-{P}_{pump}$$

where, ${P}_{net}$ is the net power, W.

## Parameters for case studies

In this case study, a downhole thermoelectric power generation system will be applied to a liquid-dominated geothermal well. The top depth of the production zone in the geothermal well is 3000 m, and the thickness of the production zone is 500 m. It was completed with a 9–5/8″ casing to the bottom. A packer was set right above the production zone with 3–1/2″ tubing connected to the surface. As designed, an injection pipe with an inner diameter of 1–5/8″ is run through the casing-tubing annulus down to the depth of 3000 m, and just penetrating the packer. The daily injection rate is 500 m^3^, and the fluid diversion ratio between the fluid entering the casing-tubing annulus above and below the packer is 1. The casing above the packer is coated with an insulation material with a thermal conductivity of 0.06 W/(m K), while the casing below the packer has a high heat conductivity with a thermal conductivity of 43.25 W/(m K) and is well cemented with formation. The tubing is also coated with an insulation material with a thermal conductivity of 0.06 W/(m K), while the injection pipe is perfectly insulated. The injected fluid will reverse flow upwards in tubing at 3500 m. Segmented thermometric generators are connected in series and fully mounted on the outer surface of the tubing above the packer. The data used in this study are summarized in Table 1.

**Table 1:** Parameters for Case Study of Downhole Thermoelectric Power Generation.

Parameters	Value	Unit
Tubing(steel) outer diameter	0.089	m
Tubing(steel) inner diameter	0.076	m
Casing(steel) outer diameter	0.244	m
Casing(steel) inner diameter	0.224	m
Injection pipe(vacuum insulated steel pipe) outer diameter	0.041	m
Injection pipe(vacuum insulated steel pipe) inner diameter	0.035	m
Wellbore depth	3500	m
Packer depth	3000	m
Coaxial heat exchanger length	500	m
Geothermal gradient	0.035	°C/m
Surface temperature	21	°C
Reservoir temperature	126	°C
Cold fluid injection temperature	20	°C
Cold fluid injection rate	500	m^3^/d
Fluid diversion ratio	1	/
Water specific heat capacity	4.18	kJ/(kg K)
Thermal conductivity of formation	2.42	W/(m K)
Thermal conductivity of insulation material (nano-SiO_2_ aerogel)	0.06	W/(m K)
Thermal conductivity of casing (steel)	43.25	W/(m K)

For downhole thermoelectric modules, Bi_2_Te_3_-based materials are selected as the semiconductor due to their commercial availability, high performance, and proven engineering applications^19^. TEG parameters are shown in Table 2. The length of each segment of TEG is assumed to be the same as the tubing length for convenient assembly and running into the downhole.

**Table 2:** Thermoelectric properties and parameters in this Case Study.

Parameters		Value	Unit
P-type: Bi_2-x_Sb_x_Te_3_	Seebeck coefficient	222.48	μV/K
	Electrical resistivity	12.5	μΩ m
	Thermal conductivity	1.36	W/(m K)
	Length	0.013	m
	Cross-section area	0.25	cm^2^
N-type: Bi_2_Se_3-y_Te_y_	Seebeck coefficient	− 223.06	μV/K
	Electrical resistivity	12.9	μΩ m
	Thermal conductivity	1.41	W/(m K)
	Length	0.013	m
	Cross-section area	0.25	cm^2^

## Results and discussion

### Results for case studies

#### Temperature distribution

**Figure 4:**
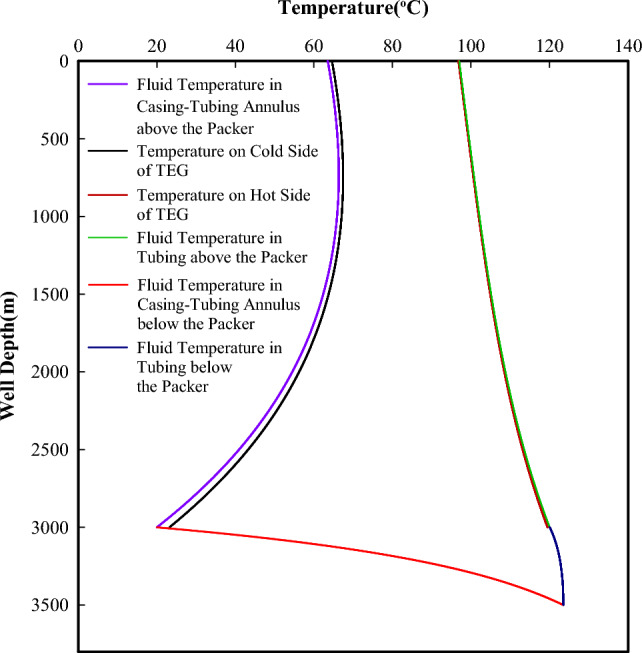
Temperature distributions in tubing, casing-tubing annulus and both sides of TEG.

The temperature distribution in the tubing, casing-tubing annulus, and hot and cold sides of TEG are presented in Fig. 4. As the fluid diverted from the downhole fluid diverter flows through the packer and enters the casing-tubing annulus, it is heated to about 123.5 °C at the well bottom. While it reverses and flows upwards in the tubing, it releases heat to the “cold” fluid in casing-tubing annulus below the packer, and the temperature of the reversed fluid at the packer drops to 120.1 °C. When the heated fluid continuously flows upwards in tubing, heat is transferred to TEG and, The reversed fluid in casing-tubing annulus above the packer, The wellhead temperature of the reversed fluid in tubing is high up to 97.0 °C, which is hot enough to be further used on the ground.

As the fluid flows upwards in the casing-tubing annulus above the packer, it is heated up by 43.5 °C due to heat conducting from the surrounding formation and transferring from TEG and tubing. The temperature differences among the reversed fluid in tubing and the hot side of TEG, the reversed fluid in casing-tubing annulus, and the cold side of TEG are relatively small. However, the temperature differences across both sides of TEG vary from 96.3 °C at the packer to 32.2 °C at the wellhead. Such temperature differences are large enough to produce electric power^24^. The continuous injection of cold fluid not only maintains a lower temperature environment in the cold side of thermoelectric module but also provides heated fluid for direct use on the ground.

#### Power generation

In this study, the net electric power from a single geothermal well is up to 228.06 kW with the proposed design. The power capacity is bit smaller in a single well, but a large benefit will be achieved when a cluster of geothermal wells is included and the reversed fluids from tubing are sent to a binary power plant as a complementary. Thermoelectric performances are listed in Table 3.

**Table 3:** Thermoelectric Performances in Case Study.

Parameters	Value	Unit
Figure of merit (Z)	0.0028	K^−1^
Dimensionless figure of merit (ZT)	0.901	/
Total produced power	320.56	kW
Required pumping power	92.50	kW
Net electric power	228.06	kW
Thermal to electricity conversion efficiency	6.63	%

#### Economical evaluation

A cost–benefit assessment was performed to evaluate the economic feasibility of downhole thermoelectric power generation in a geothermal well. Here, we assume that the geothermal well has already existed; what we only need to do is retrofit the well to be suitable for power generation. Therefore, only the capital cost of the TEG system installation and the circuitry construction are considered in the cost–benefit assessment. In addition, the power generation by geothermal energy has a large environmental benefit. More than 90% of greenhouse gas emissions could be reduced if the electrical energy produced from a fossil fuel power plant is replaced by geothermal energy^55^. Therefore, the carbon tax income is included in the cost–benefit assessment.

According to the dimensions of P–N type semiconductors and the length and diameter of tubing, a total of 126,000 thermoelectric modules (each module is made up of 16 × 16 P–N type semiconductors) are needed to be mounted on the outer surface of tubing. Assuming the cost of a thermoelectric module is 35 RMB, the costs of accessories and installations are 25% of the total cost of thermoelectric modules^56^. The cost parameters are listed in Table 4.

**Table 4:** Capital cost of a downhole thermoelectric power generation system.

Parameters	per Price (RMB)	Quantity	Total Cost (× 10^4^RMB)
Thermoelectric module	35	126,000	441
Voltage regulator system	2,500	20	5
Electric storage system	/	/	200
Accessories and installations	/	/	110.25
Total capital cost			756.25

Electricity generation from geothermal resources results in much lower greenhouse gas (GHG) emissions than those from traditional fossil fuels^57^. According to the report by the National Renewable Energy Laboratory (NREL), the median life cycle GHG emissions from enhanced geothermal systems binary, hydrothermal flash, and hydrothermal binary plants were found to be 32 g CO_2_ eq/kWh, 47 g CO_2_ eq/kWh, and 11.3 g CO_2_ eq/kWh respectively^58^. For power generation from coal-fired plants, the median life-cycle GHG emission is estimated to be 1018 g CO_2_ eq/kWh^59^. If the average median life cycle GHG emission from geothermal resources is taken as 30 g CO_2_ eq/kWh, the reduction of GHG emissions is 988 g CO_2_ eq/kWh when the electricity generated from a coal-fired plant is replaced by a geothermal power plant. It is assumed that a carbon tax will be imposed on power plants in the future. Supposing carbon tax is 20–200 RMB/t CO_2_^59^, then an additional income of a geothermal power plant will increase by 0.0198–0.1976 RMB/kWh. The parameters used for cost–benefit analysis are shown in Table 5, and the results for different carbon tax scenarios are presented in Table 6.

**Table 5:** Parameters used for cost–benefit analysis.

Parameters	Value	Unit
Electric price	1	RMB/kWh
Operation life	18	Year
Annual operation time	6000	Hours
Fixed depreciation period	10	Year
Income tax	25	%
Benchmark return on investment	9	%
Base rate of return	6	%
Discount rate	6	%

**Table 6:** Results of cost–benefit analysis for different carbon tax scenarios.

Parameters	Carbon Tax(RMB/t CO_2_)		
	0	20	200
Annual net income(10^4^RMB)	121.53	123.57	141.81
Rate of return(%)	16.07	16.34	18.75
Static payback period(Year)	6.22	6.12	5.33
Dynamic payback period (Year)	8.02	7.85	6.62
Net present value(10^4^RMB)	559.66	581.67	779.24

It can be seen that the investment could be returned in 6 to 8 years, and the rate of return is greater than 16% for different carbon tax scenarios. Based on the above calculation, downhole thermoelectric power generation with a coaxial borehole heat exchanger is feasible and economically competitive.

### Flow rate analysis

**Figure 5:**
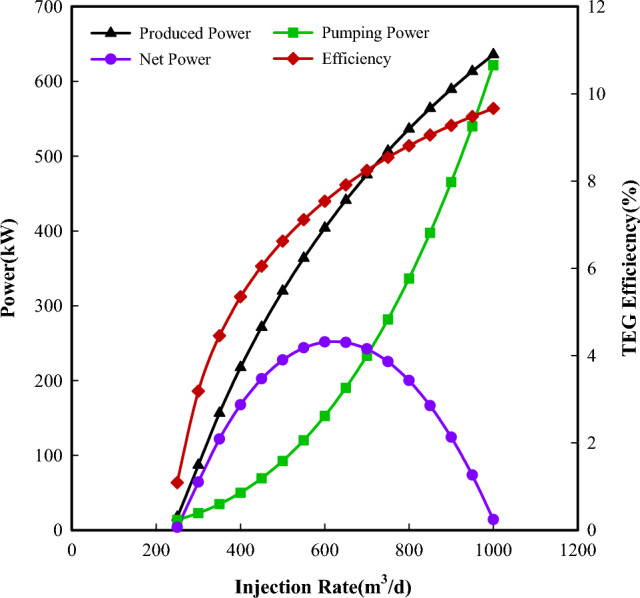
Powers and efficiency change with injection rate.

Based on the proposed configuration and taking the fluid diversion ratio as 1, the power and efficiency of the system as a function of injection rate were calculated and shown in Fig. 5. From this figure, the required pumping power has an approximate exponential relationship with the injection rate. The increased injection rate results in an increase of friction loss in the injection pipe, and a larger pumping power is required to ensure fluid circulated in the system. Meanwhile, the increased flow velocity leads to an increase of Reynolds number and Prandtl number, which accelerate the heat transfer between the reversed fluid in the tubing and the hot side of TEG and between the reversed fluid in the casing-tubing annulus and the cold side of TEG. The enhanced heat transfer improves the temperature differences across both sides of TEG and results in a gradual increase in the power produced. But the increments of produced power are slower than the required pumping power. Therefore, the net power shows an increasing and then decreasing change with the increasing injection rate. The maximum net power is achieved around the injection rate of 600 m^3^/d. The power efficiency shows the same variation as the produced power.

**Figure 6:**
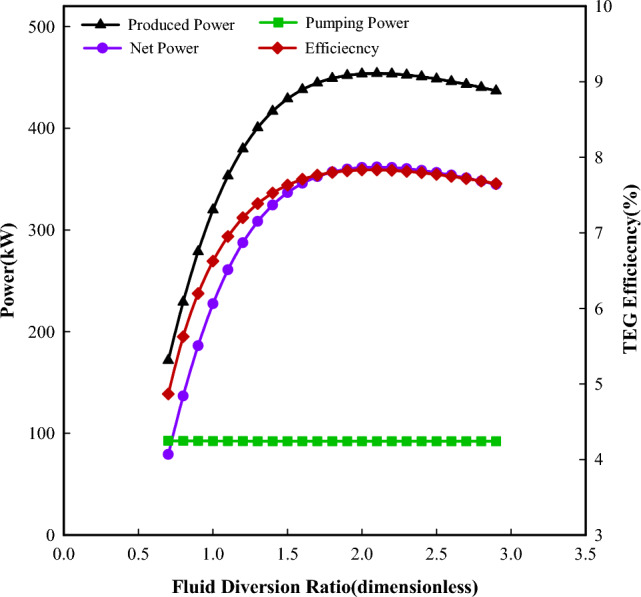
Powers and efficiency change with fluid diversion ratio at a injection rate of 500 m^3^/d.

Figure 6 shows the power and efficiency of the system as a function of the fluid diversion ratio at an injection rate of 500 m^3^/d. With the increasing fluid diversion ratio, the reversed fluid in the casing-tubing annulus increases and the reversed fluid in the tubing decreases. More fluids flowing in the casing-tubing annulus are beneficial to keep the cold side of TEG at a lower temperature, but the potential of lowering the temperature on the cold side of TEG by increasing the flow rate in the casing-tubing annulus may be limited. Meanwhile, the decreased fluid flowing into the coaxial borehole heat exchanger will result in less heat harvesting from the surrounding formation and lower the fluid temperature in the tubing at the packer. Therefore, the temperature difference across both sides of the TEGs shows an increasing and then decreasing change with increasing fluid diversion ratio. Thus, the produced power begins to decrease gradually after the fluid diversion ratio is higher than a certain value. The required pumping power shows a smaller change, which indicates that the friction loss may be mainly consumed in the injection pipe. The thermal-to-electricity conversion efficiency has the same variation as the produced power and the net power. From the results, the reasonable fluid diversion ratio is between 1.5 and 2.

### Flow conduit analysis

**Figure 7:**
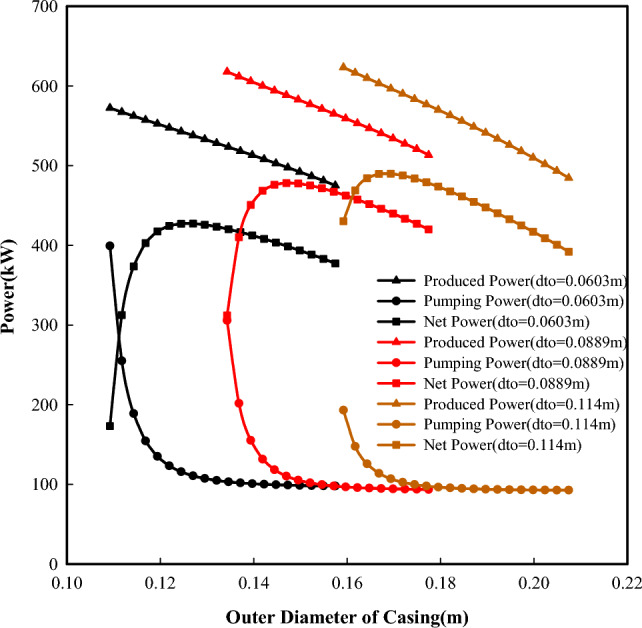
Powers change with different tubing-casing configurations.

**Figure 8:**
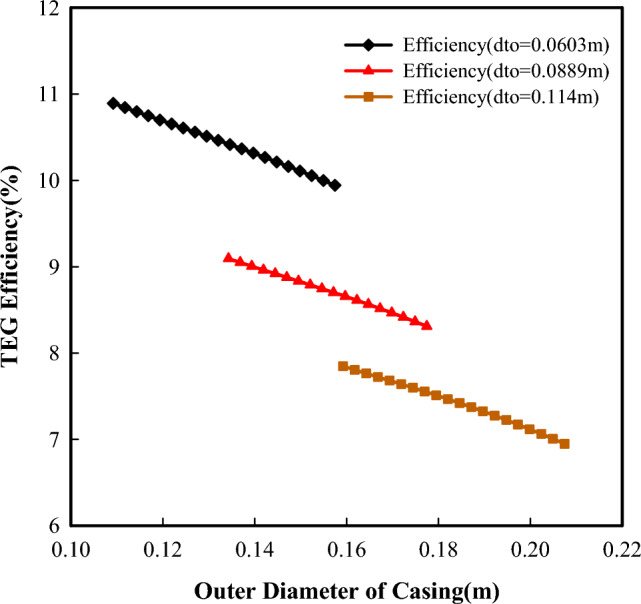
Efficiency changes with different tubing-casing configurations.

**Figure 9:**
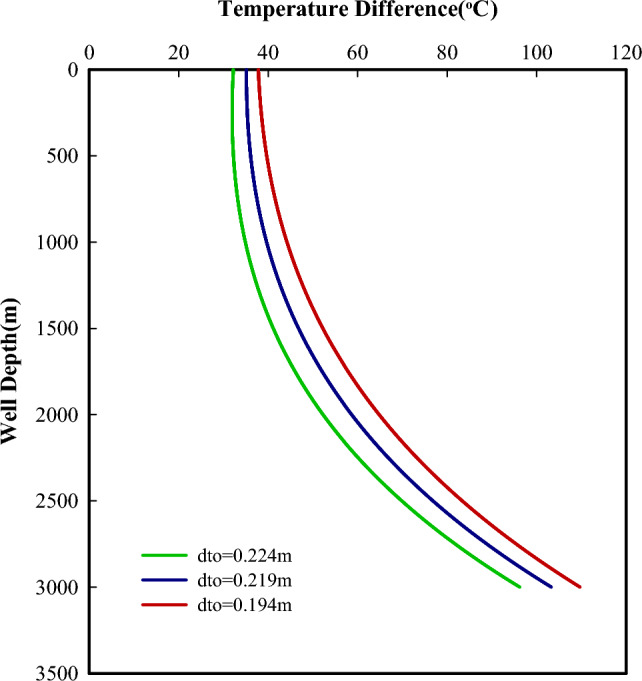
Temperature differences across both sides of TEGs with a constant tubing size (0.0889m).

The size of the flow conduit is an important factor that affects the flow velocity of the fluid, the heat transfer process, and friction losses. Figures 7 and 8 present the powers the efficiency of the system as a function of tubing-casing configurations. For a constant tubing size,a smaller casing will have a smaller casing-tubing annulus, which will result in a faster flow velocity of the reversed fluid in it. Thus, more heat will be taken away from the cold side of TEGs. This is helpful to keep the cold side of TEGs in a lower temperature condition and further increase the temperature difference across both sides of TEGs (Fig. 9), which will produce more power with the fixed number of thermoelectric modules. It can be seen that the power produced decreases with increased casing size. With the increasing casing-tubing annulus, the changes in flow velocity and the fiction losses become slower and smaller, and the required pumping power becomes stable after a gradual decrease. Therefore, the net power shows an increasing and then decreasing variation with increased casing size.

**Figure 10:**
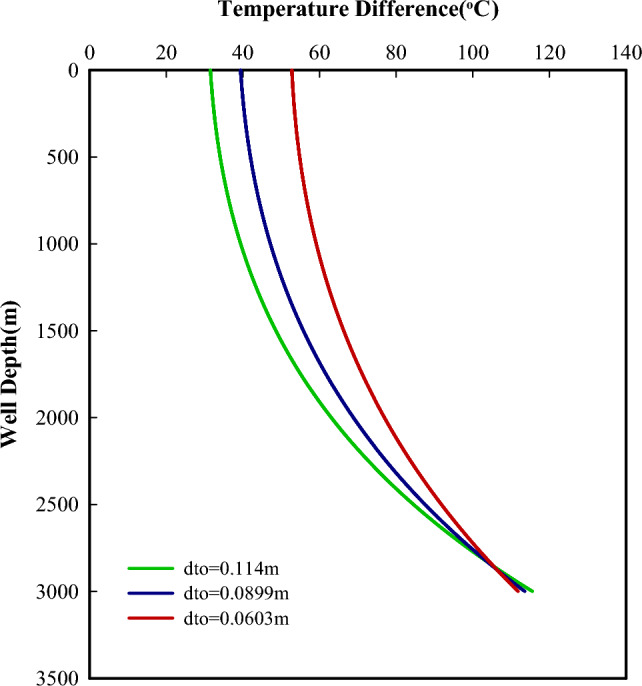
Temperature differences across both sides of TEGs with a constant casing size (0.176m).

For a constant casing size, smaller tubing will result in a faster flow velocity in the tubing. This makes the reversed fluid in the tubing being transfer more heat to the hot side of TEG. It is helpful to keep the hot side of TEG at a higher temperature and further a larger temperature difference across both sides of TEGs (Fig. 10), which may produce more power according to the thermoelectric principle. However, the power produced increases with increased tubing size (Fig. 9). This is directly related to the number of thermoelectric modules, as the increased tubing outer surface will accommodate more thermoelectric modules. When the tubing sizes are 0.0603 m, 0.0889 m and 0.114 m, the number of thermoelectric modules is 84,000, 126,000, and 165,000, respectively. From this point, the tubing size (i.e., the number of thermoelectric modules) outweighs the temperature difference across both sides of TEGs in term of produced power.

## Conclusions

We propose a novel design for downhole power generation, integrating thermoelectric technology with a coaxial borehole heat exchanger. This innovative approach harnesses the temperature differentials between the reversed fluid in the casing-tubing annulus, redirected by the downhole fluid diverter, and the reversed fluid in the tubing from the coaxial borehole heat exchanger (specifically, the casing-tubing annulus below the packer) to facilitate downhole power generation. The established models provide a comprehensive understanding of temperature distributions in the casing-tubing annulus, tubing, coaxial borehole heat exchanger, and both sides of the thermoelectric generator (TEG). Additionally, a case study for a geothermal well was conducted, shedding light on the system's performance under various parameters such as injection rate, fluid diversion ratio, and casing-tubing configuration. This innovative design not only showcases the feasibility of downhole power generation but also underscores the importance of optimizing key parameters for enhanced system efficiency. The proposed approach holds significant potential for advancing the utilization of geothermal energy in a sustainable and economically viable manner. The conclusions are drawn as follows:

1. Downhole thermoelectric power generation with a coaxial borehole heat exchanger in geothermal wells is a new way to utilize geothermal energy in the downhole without affecting subsequent utilization on the ground. In a geothermal well with a depth of 3500 m including the 500 m length of the heat exchange zone, a net power output of 228.06 kW and thermal-to-electricity conversion efficiency of 6.63% are obtained. The payback period under different carbon tax scenarios is about 6–8 year, and the rate of return is higher than 16%.
2. The Injection rate and fluid diversion ratio will influence the produced power, required pumping power, and thermal-to-electricity conversion efficiency. A larger flow rate will accelerate the heat transfer process and maintain a larger temperature difference across both sides of TEG. The reasonable power and efficiency will be obtained with an injection rate of 600 m^3^/d and a fluid diversion ratio of 1.5–2.0.
3. The sizes of casing-tubing configurations affect the heat transfer between the fluids and TEGs. The effect of the tubing size on the produced power is greater than the casing-tubing annulus and temperature gradient across both sides of TEG because a larger tubing size could accommodate more thermoelectric modules.
4. A higher temperature difference is crucial for better downhole thermoelectric performance. The Proposed downhole thermoelectric power generation design with a coaxial borehole heat exchanger is also applicable for abandoned geothermal wells, enhanced geothermal systems, and abandoned oil and gas wells.

## Data Availability

The data that supports the findings of this study is available from the corresponding author upon reasonable request.
